# Supplementary material for: Left-wing support of authoritarian submission to protect against societal threat
Source: PLoS One. 2022 Jul 19;17(7):e0269930. doi: 10.1371/journal.pone.0269930 (PMC9295988; doi:10.1371/journal.pone.0269930)
Supplement: S1 Appendix — (DOCX) [file pone.0269930.s001.docx]

**Appendix A**

Fear of COVID-19 scale items

The scale consists of 7 items and participants responded using a 5-point Likert scale ranging from 1 (*strongly disagree*) to 5 (*strongly agree*).

1. I am most afraid of the coronavirus
2. It makes me uncomfortable to think about coronavirus
3. My hands become clammy when I think about coronavirus
4. I am afraid of losing my life because of coronavirus
5. I become nervous or anxious when watching news and stories about coronavirus
6. I cannot sleep because I am worried about getting coronavirus
7. My heart races or palpitates when I think about coronavirus
