# Supplementary material for: Left-wing support of authoritarian submission to protect against societal threat
Source: PLoS One. 2022 Jul 19;17(7):e0269930. doi: 10.1371/journal.pone.0269930 (PMC9295988; doi:10.1371/journal.pone.0269930)
Supplement: S2 Appendix — (DOCX) [file pone.0269930.s002.docx]

**Appendix B**

Aggression – Conservatism – Traditionalism (ACT) scale items

The scale consisted of 36 items split evenly across three subscales. Participants responded using 7-point Likert scales ranging from 1 (*very strongly disagree*) to 7 (*very strongly agree*).

**Aggression (Authoritarianism)**

1. Strong, tough government will harm not help our country.
2. Being kind to loafers or criminals will only encourage them to take advantage of your weakness, so it’s best to use a firm, tough hand when dealing with them.
3. Our society does NOT need tougher government and stricter laws.
4. The facts on crime and the recent public disorders show we have to crack down harder on troublemakers, if we are going preserve law and order.
5. Our prisons are a shocking disgrace. Criminals are unfortunate people who deserve much better care, instead of so much punishment.
6. The way things are going in this country, it’s going to take a lot of “strong medicine” to straighten out the troublemakers, criminals, and perverts.
7. We should smash all the negative elements that are causing trouble in our society.
8. The situation in our country is getting so serious, the strongest methods would be justified if they eliminated the troublemakers and got us back to our true path.
9. People who say our laws should be enforced more strictly and harshly are wrong. We need greater tolerance and more lenient treatment for lawbreakers.
10. The courts are right in being easy on drug offenders. Punishment would not do any good in cases like these.
11. What our country really needs is a tough, harsh dose of law and order.
12. Capital punishment is barbaric and never justified.

**Submission (Conservatism)**

1. It’s great that many young people today are prepared to defy authority.
2. What our country needs most is discipline, with everyone following our leaders in unity.
3. Students at high schools and at university must be encouraged to challenge, criticize, and confront established authorities.
4. Obedience and respect for authority are the most important virtues children should learn.
5. Our country will be great if we show respect for authority and obey our leaders.
6. People should be ready to protest against and challenge laws they don’t agree with.
7. People should be allowed to make speeches and write books urging the overthrow of the government.
8. The more people there are that are prepared to criticize the authorities, challenge and protest against the government, the better it is for society.
9. People should stop teaching children to obey authority.
10. The real keys to the “good life” are respect for authority and obedience to those who are in charge.
11. The authorities should be obeyed because they are in the best position to know what is good for our country.
12. Our leaders should be obeyed without question.

**Conventionalism (Traditionalism)**

1. Nobody should stick to the “straight and narrow.” Instead people should break loose and try out lots of different ideas and experiences.
2. The “old-fashioned ways” and “old-fashioned values” still show the best way to live.
3. God’s laws about abortion, pornography, and marriage must be strictly followed before it is too late.
4. There is absolutely nothing wrong with nudist camps.
5. This country will flourish if young people stop experimenting with drugs, alcohol, and sex, and pay more attention to family values.
6. There is nothing wrong with premarital sexual intercourse.
7. Traditional values, customs, and morality have a lot wrong with them.
8. Everyone should have their own lifestyle, religious beliefs, and sexual preferences, even if it makes them different from everyone else.
9. The radical and sinful new ways of living and behaving of many young people may one day destroy our society.
10. Trashy magazines and radical literature in our communities are poisoning the minds of our young people.
11. It is important that we preserve our traditional values and moral standards.
12. People should pay less attention to the bible and the other old-fashioned forms of religious guidance, and instead develop their own personal standards of what is moral and immoral.
