# Supplementary material for: Left-wing support of authoritarian submission to protect against societal threat
Source: PLoS One. 2022 Jul 19;17(7):e0269930. doi: 10.1371/journal.pone.0269930 (PMC9295988; doi:10.1371/journal.pone.0269930)
Supplement: S1 Table — (DOCX) [file pone.0269930.s003.docx]

**Table S1.** Descriptive statistics of demographic variables.

|  | M | SD |
| --- | --- | --- |
| Sex (% Female) | 0.40 |  |
| Age | 47.54 | 16.24 |
| Political beliefs | 3.74 | 1.48 |
| National support | 0.45 | 3.17 |
| Labour Support | -0.43 | 3.30 |
