# Supplementary material for: Left-wing support of authoritarian submission to protect against societal threat
Source: PLoS One. 2022 Jul 19;17(7):e0269930. doi: 10.1371/journal.pone.0269930 (PMC9295988; doi:10.1371/journal.pone.0269930)
Supplement: S2 Table — (DOCX) [file pone.0269930.s004.docx]

**Table S2.** Outcome of confirmatory factor analysis for parcelled ACT scale.

| Latent Variable | Parcel | Estimate | Standard Error | z-score | p-value |
| --- | --- | --- | --- | --- | --- |
| Submission | C_1 | 0.82 | 0.00 |  |  |
|  | C_2 | 0.83 | 0.02 | 36.33 | <0.01 |
|  | C_3 | 0.79 | 0.03 | 33.87 | <0.01 |
|  | C_4 | 0.87 | 0.02 | 38.63 | <0.01 |
|  |  |  |  |  |  |
| Conventionalism | T_1 | 0.81 | 0.00 |  |  |
|  | T_2 | 0.88 | 0.03 | 39.18 | <0.01 |
|  | T_3 | 0.84 | 0.03 | 36.69 | <0.01 |
|  | T_4 | 0.76 | 0.03 | 31.80 | <0.01 |
|  |  |  |  |  |  |
| Aggression | A_1 | 0.70 | 0.00 |  |  |
|  | A_2 | 0.87 | 0.05 | 31.72 | <0.01 |
|  | A_3 | 0.77 | 0.04 | 28.14 | <0.01 |
|  | A_4 | 0.85 | 0.05 | 31.08 | <0.01 |
|  |  |  |  |  |  |
| RWA | Submission | 0.58 |  |  |  |
|  | Conventionalism | 0.64 |  |  |  |
|  | Aggression | 0.78 |  |  |  |
