# Supplementary material for: Left-wing support of authoritarian submission to protect against societal threat
Source: PLoS One. 2022 Jul 19;17(7):e0269930. doi: 10.1371/journal.pone.0269930 (PMC9295988; doi:10.1371/journal.pone.0269930)
Supplement: Attachment — Submitted filename: feedback-ds1.docx [file pone.0269930.s005.docx]

**Reviewer #1**

The first issue is empirical. Because support for the Labour Party and support for the National Party were negatively associated with each other (correct? I don't see anywhere in this paper a zero-order correlation matrix of all the variables, something that should always be presented in a paper reporting a study of individual differences), yet both were positively related to authoritarian submission, the question arises: what were the political leanings of the respondents who scored *low* on authoritarian submission? Were they middle-of-the-roaders, with opinions in between Labour and National? Were they adherents of parties to the left of Labour? To the right of National? Or were they adherents of parties with platforms orthogonal to the traditional left-right dimension? It appears that the authors can't answer this key question, because they didn't collect enough data. They state that 24% of NZ's voters cast ballots for parties other than Labour or National in the 2020 election. For a study of political psychology, that's a large percentage of your population to leave without the opportunity to express their political preference.

*We agree with the reviewer that a correlation matrix could contribute to scene setting prior to our analyses to address some of the questions raised here. This can now be found at the beginning of our results section with some written interpretation on findings. Regarding the party support questions used in the study, there are two reasons we believe its appropriate even if 24% of people voted for other parties: (1) The New Zealand political system is less polarised than countries such as the US so supporting a major party on some level despite voting for a minority party would not be unusual (you expect your minority party to form a coalition or confidence in supply agreement with a majority party). (2) Our second analysis used political orientation and the results aligned with our findings when using political party support.*

The second issue has to do with the construct of Left-Wing Authoritarianism (LWA), which the authors dismiss with a few sentences in the Discussion. First, although the Manson (2020) study is relevant, the key paper here (already cited 38 times according to Google Scholar), yet which the authors inexplicably fail to cite, is:

Costello, T. H., Bowes, S. M., Stevens, S. T., Waldman, I. D., Tasimi, A., & Lilienfeld, S. O. (2021). Clarifying the structure and nature of left-wing authoritarianism. Journal of Personality and Social Psychology.

This is a solid, thorough, multi-study work of political psychometrics, that (in my view) firmly establishes LWA as a valid construct. The authors of the present study must engage with it seriously, and they must admit that a major limitation of their own study is that they did not measure LWA.

*We agree with the reviewer that this paper was a significant contribution to the LWA literature. Due to significant delays during the review process, this study was not actually published when the current manuscript was submitted. We now discuss the Constello et al. paper in the Discussion.*

A third issue, which I wouldn't raise if the authors had stuck to description and not ventured into editorializing (lines 290-292), is their repeated, uncritical description of COVID-19 as an "existential threat" that necessitated NZ's lockdown measures. Was a virus with a 0.3% case fatality rate (at its worst) really an existential threat? Sweden imposed only mild restrictions on business and social life, and not only does it still exist, its per-capita COVID death rate wasn't among the highest in Europe. If you're going to editorialize about the lockdowns being a "good thing," then you need to address the complicated cost-benefit analysis entailed by them. The lockdowns caused substantial harms (economic, psychological, and even medical in the form of delayed cancer screenings and so forth) that must be balanced against their benefits in slowing the spread of COVID. But all that would be beyond the scope of this paper. Instead, I suggest deleting the sentence in lines 290-292, and changing the phrase "existential threat" to "perceived existential threat" throughout this paper.

*We thank the reviewer and have omitted this sentence. We have also made it clear that we are referring to a perceived existing threat. It is also important to note that, when the current study was conducted, the world had little knowledge on the pandemic beyond knowing it was deadly and contagious, as was being witnessed overseas at the time.*

Minor issues of mistaken or missing words:
Line 65, a word is missing between "spectrum" and "the"
Line 271: shouldn't that be "aggression" rather than "submission"?
Line 250: shouldn't that be "aggression" rather than "authoritarianism"?

*We thank the reviewer for identifying these errors and have corrected them.*

**Reviewer #2**

The first – and maybe most serious – one relates to the process of data collection. The paper is generally very short on this issue, but it seems that participants to the survey were recruited via online news websites and social media (Facebook), i.e. there is neither quota sampling nor genuine random sampling. The chosen strategy is very likely to create a severely biased sample. While it is perhaps permissible to employ this snow-balling technique of recruitment for pre-tests and – exceptionally – for survey experiments, I am highly doubtful that it can yield reliable estimates for surveys that at least have the aim of approaching representativeness. A thorough description of the distributions of core variables in the survey sample and a comparison with existing population data could have mitigated (or exacerbated?) some of these concerns. In any case, this fundamental flaw in the data collection process would be a “no-go” in high-ranked peer reviewed journals in political science and sociology.

*We thank the reviewer for their feedback and seek to clarify our recruitment method. Although Facebook recruitment alone could be biased, the online news websites we utilised did represent a broad political spectrum. Furthermore, we would suggest that the findings would still hold interest even if they were biased. This is due to the juxtaposition in submission showing a positive relationship with Labour party support despite aggression and traditionalism holding a negative relationship with Labour party support. On that note, we have added a limitation describing this nuance and will seek to generate a complex survey sample with adequate post-stratification when we seek to progress this line of research in the future.*

The second major point is the fact that the paper is interested in how the Covid-19 pandemic has (potentially) changed authoritarian attitudes. A significant limitation in that regard (which the authors fully acknowledge) is that they do not have data on these attitudes from before the crisis (or – for obvious reasons – after the crisis). The authors mention a study that could potentially serve as anchoring point, but they do not actually discuss the findings of this study in detail (which seems to be a pre-study anyway). Hence, the central ambition of the paper – to study the effect of the crisis on changing attitudes – can simply not be met. Therefore, the framing of the paper should be adjusted and toned down in that regard. Realistically, it can also study the association between ideology and authoritarian attitudes in the context of the crisis at a specific point in time.

*We agree with the reviewer that it could be misleading to indicate the present paper makes some inference over time and the focus is the surprising difference between subfactors. We have adjusted the manuscript to make it clear that the core finding is in regard to the latter.*

A third issue is about the interpretation of the core finding and the associated theoretical mechanism. The core finding – as I see it – is that the degree of “authoritarian submission” (related to support for Covid-related policy measures) did not differ significantly between supporters from different parties at the height of the first lockdown. The paper interprets this as indicated wide-spread support for authoritarian values in the face of crisis across the board, i.e. the crisis threat overwhelms potential concerns that left-wing people might have against lockdown measures. However, the paper does not take into account a potential “rally behind the flag”-effect that is quite well-known in other domains (e.g. in the case of wars). In crisis moments, citizens tend to rally behind their political leadership, in particular left-wing respondents could become more supportive of whatever “their” left-wing government is doing. The mechanism is then, however, not a changing ideology, but party loyalty.

*We agree with the reviewer and have included detail on this particular effect as it relates to our findings.*

Besides these major points, I have a few smaller issues:
- The data and the regression analysis work with a rather reduced set of socio-economic control variables. What is in particular missing is information about respondents’ educational background, their socio-economic position, their income, and potentially their labor market position. These are quite standard things to control for in studies of attitudes and preferences in political science, so I was surprised to not find them here.
- It would be good to have the detailed wordings of all the items in both the scales on authoritarianism and fear of Covid, at least in the appendix.
- The paper would benefit from putting the case of New Zealand into a more comparative context. What kind of broader implications can researchers draw from studying this relatively peculiar and special case?

*We thank the reviewer for their feedback and have added the requested appendices for clarity. Unfortunately, we did not collect the socioeconomic variables requested, as is more typical in the field of psychology, but will endeavour to include a richer variety of covariates in future political science focused research.*
