# Supplementary material for: Left-wing support of authoritarian submission to protect against societal threat
Source: PLoS One. 2022 Jul 19;17(7):e0269930. doi: 10.1371/journal.pone.0269930 (PMC9295988; doi:10.1371/journal.pone.0269930)
Supplement: Attachment — Submitted filename: r4_feedback.docx [file pone.0269930.s006.docx]

Dear Editor,

We thank the editorial team and reviewers for an opportunity to make minor revisions to our manuscript. As seen below, we have addressed all comments raised by the editor and reviewer one.

**Editor**

- Journal Requirements:

  Please review your reference list to ensure that it is complete and correct. If you have cited papers that have been retracted, please include the rationale for doing so in the manuscript text, or remove these references and replace them with relevant current references. Any changes to the reference list should be mentioned in the rebuttal letter that accompanies your revised manuscript. If you need to cite a retracted article, indicate the article’s retracted status in the References list and also include a citation and full reference for the retraction notice.

*We have made additions to the reference list included below.*

- Please also consider other papers to focus on the economic effects on populism, such as

  Gozgor, G. (2022). The role of economic uncertainty in the rise of EU populism. Public choice, 190(1), 229-246.
  Guriev, S. (2018). Economic drivers of populism. American Economic Review Papers and Proceedings, 108(5), 200–203.
  Margalit, Y. (2019). Economic insecurity and the causes of populism, reconsidered. Journal of Economic Perspectives, 33(4), 152–170.
  Rodrik, D. (2018). Is populism necessarily bad economics? American Economic Review Papers and Proceedings, 108(5), 196–199.
  Rodrik, D. (2021). Why does globalization fuel populism? Economics, culture, and the rise of right-wing populism. Annual Review of Economics, 13, 133–170.

*We thank the reviewer for raising this nuance to be included in the manuscript on lines 326 to 329 where we now also cite Gozgor (2022), Guriev (2018), and Margalit (2019).*

- Thank you for including your ethics statement on the online submission form: 

  "Ethics was granted by the University of Otago Human Ethics Committee. Consent was granted in writting, and, due to the sensitive nature of COVID-19 at the time of collection. Participants separately consented to answer COVID-19 related questions."

  To help ensure that the wording of your manuscript is suitable for publication, would you please also add this statement at the beginning of the Methods section of your manuscript file.

*We now include this statement in the method section.*

**Reviewer 1**

- This revision is almost ready for publication. I suggest restoring the reference to the Manson (2020) paper, by adding the following sentence, in line 322, after the sentence that ends "...while the remaining components are unique":
  "With regard to COVID-19 mitigation specifically, Manson (##), using a short version of Costello, Bowles' (##) LWA scale, found that Americans high in either LWA or RWA were more likely, in Spring 2020, to favor many of the same intrusive government mitigation policies, compared to individuals who scored low in both forms of authoritarianism."

*We thank the reviewer for their feedback and now include this after line 322 along with the Manson study.*
